# Being tolerated: Implications for well‐being among ethnic minorities

**DOI:** 10.1111/bjop.12492

**Published:** 2021-02-11

**Authors:** Sara Cvetkovska, Maykel Verkuyten, Levi Adelman, Kumar Yogeeswaran

**Affiliations:** ^1^ ERCOMER Utrecht University Netherlands; ^2^ University of Canterbury New Zealand

**Keywords:** acceptance, discrimination, minorities, tolerance, well‐being

## Abstract

Tolerating or condoning practices that one finds objectionable is typically considered a positive way to negotiate intergroup differences. However, being the target of tolerance might harm well‐being, which we examined in three studies (a survey and two experiments) among a total of 1,054 members of various racial/ethnic minority groups in the United States. In Study 1, we found that perceiving oneself to be tolerated on the basis of one’s ethnic group membership was associated with more negative well‐being. In Study 2, we found that bringing to mind experiences of being tolerated results in less positive and more negative affect than thinking about experiences of acceptance, but more positive and less negative outcomes than thinking about overt discrimination experiences. In Study 3, we replicated the results of Study 2 while demonstrating that threat to social identity needs mediates the tolerance–well‐being link. These results suggest that being tolerated is related to minority targets’ well‐being in ways that are intermediate between being treated with outright discrimination and full acceptance, but that being tolerated follows a pattern closer to discrimination.

## Background

Many nations and civil organizations increasingly promote tolerance as a condition for multicultural justice and positive intergroup relations in plural societies (Verkuyten, Yogeeswaran, & Adelman, 2019). It is clear that there is no space for diversity and difference without the capacity to put up with practices that one finds objectionable: ‘diversity and equality among people living in peace *necessarily* means that they have learned to tolerate one another’ (Vogt, 1997, p. 5, original italics). Tolerance implies forbearance and putting up with something that one disagrees with, disapproves of, or is negative about (Cohen, 2004). Thus, tolerance differs from indifference and being non‐judgemental because ‘one cannot tolerate ideas of which one approves’ (Gibson, 2006, p. 22). In tolerance, one decides to endure dissenting beliefs or practices for other reasons, such as the endorsement of egalitarianism (Cohen, 2004; Verkuyten & Yogeeswaran, 2017). While people cannot be expected to appreciate all different practices and beliefs, especially when these clash with one’s own convictions, they may be able to show forbearance and tolerate these differences.

However, tolerance implies that minority group members are allowed to engage in practices that tolerators consider misguided, offensive, or wrong. Tolerance carries ‘echoes of at best grudging acceptance, and at worst ill‐disguised hostility’ (Fitzgerald, 2000, p. 13) and involves ‘the marking of subjects of tolerance as inferior, deviant, or marginal vis‐à‐vis those practicing tolerance’ (Brown, 2006, p. 13). Furthermore, tolerance places targets in a vulnerable position wherein their freedom can be limited when more powerful others consider them no longer tolerable (Verkuyten, Yogeeswaran, & Adelman, 2020b, 2020a). Thus, although the act of tolerating others may be critical for managing diversity in a way that permits contradictory beliefs and values to coexist in society, the experience of being tolerated may be less positive. Tolerance might create negative psychological consequences for tolerated individuals, who typically want to be valued and respected (Bergsieker, Shelton, & Richeson, 2010) rather than *merely* tolerated (Parekh, 2000).

In this paper, we present three studies conducted in the United States that examine the implications of being tolerated on the well‐being of racial/ethnic minority group members. In doing so, we compare the implications of being tolerated with being fully accepted and being overtly discriminated against. This allows us to examine the claim that ‘tolerance involves an attitude that is intermediate between wholehearted acceptance and unrestrained opposition’ (Scanlon, 2003, p. 187) from the target’s perspective. Being tolerated implies that one has the opportunity to live as one wants and enjoy the psychological benefits of this, but may also harm one’s well‐being due to the precariousness and disapproval involved in tolerance.

### Being tolerated and well‐being

There is a growing body of research providing evidence for a distinction between tolerance, acceptance, and discrimination on the part of the tolerators (e.g., Verkuyten, Yogeeswaran & Adelman, 2020b, 2019; Verkuyten & Yogeeswaran, 2017). While discrimination implies that one cannot live with a particular group or practice (such as the wearing of a headscarf by Muslim women), tolerance involves the feeling of ‘I can live with it’, while acceptance moves in the direction of ‘It is welcome’. However, there is little research into the experience of being tolerated and how this might differ from being accepted and being discriminated against. Furthermore, while the harmful effects of discrimination on targets’ well‐being have been well documented (Pascoe & Richman, 2009; Schmitt, Branscombe, Postmes, & Garcia, 2014), there is hardly any theorizing and research on the consequences of being tolerated. One exception is survey research among Dutch ethnic minorities that found that the perception of being tolerated was related to more positive affect compared to discrimination (Cvetkovska, Verkuyten, & Adelman, 2020). Yet, the condescension and conditionality of tolerating minority practices are also likely to harm targets’ overall well‐being and this may be due to threats to minority members’ social identity needs.

Theoretically, tolerance is argued to entail several unintended negative consequences by undermining minority targets’ social identity needs (Verkuyten et al., 2020b, 2020a). Due to the implied devaluation of minority practices and beliefs, tolerance may feel like a condescending orientation which could harm minorities’ self‐esteem and evoke negative emotions associated with rejection. Furthermore, being tolerated may threaten minority targets’ sense of control, as tolerators retain the power to decide the terms under which targets may practice their way of life. Thus, the satisfaction of social identity needs such as self‐esteem, belonging, and efficacy (Vignoles, 2011) may be undermined among the tolerated.

Initial evidence for the role of threatened identity needs comes from a survey study conducted among disabled, LGBTQ+, and Kurdish people in Turkey (Bagci et al., in press). Independently of perceived discrimination, it was found that higher perceived toleration was related to threatened identity needs which, in turn, were related to lower well‐being in all three minority groups. However, this study did not compare being tolerated to being fully accepted, did not allow for causal inferences due to its correlational nature, and did not account for critical individual difference factors such as one's general tendency to feel negative emotions.

Although group‐based treatment can affect well‐being in general, several facets of well‐being might be especially affected by perceiving oneself to be tolerated on the basis of minority group membership. For instance, the disapproval of tolerance might give rise to feelings of offence, discomfort, and irritation. The conditionality of tolerance could also evoke fear, uncertainty, and vigilance, which are taxing to targets’ well‐being (Verkuyten et al., 2020b, 2020a). More reflective dimensions of well‐being can also be affected by the perceived treatment of one’s minority group. For example, discrimination has been shown to decrease the overall life satisfaction of African Americans (Broman, 1997; Yap, Settles, & Pratt‐Hyatt, 2011). Tolerance conveys that one can only act freely under conditions set by tolerators more powerful than oneself. Therefore, the feeling of being merely tolerated might also be associated with lower life satisfaction.

Tolerance may also impact upon self‐directed affect, such as self‐esteem. Knowing that others hold objections to core parts of one’s identity conveys negative public regard (Leary & Baumeister, 2000; Schmitt et al., 2014; Sellers, Smith, Shelton, Rowley, & Chavous, 1998). Negative consequences for self‐esteem would be especially likely for members of disadvantaged groups and for concealable stigmas (Schmitt et al., 2014). Because tolerance involves disapproval of the target’s actions, targets might want to avoid negative evaluations by refraining from engaging in certain practices and conceal stigmatized self‐aspects. Such attempts to avoid others’ negative reactions have been shown to harm self‐directed affect (Barreto, Ellemers, & Banal, 2006).

Being tolerated might also have a negative impact on one’s sense of control. A sense of control over one’s life is considered to be a key component of well‐being (Ryan & Deci, 2017), which is most clearly thwarted by experiencing pervasive discrimination, as this implies that more powerful others control important outcomes in one’s life. Being tolerated may also be experienced as having less control, as it is a more powerful other that decides the conditions and limits of what one is allowed to do. Thus, even though the tolerating group refrains from negatively interfering in the lives of the tolerated, tolerance does not provide the conditions for minority group members to be free from domination. A more powerful outgroup dictates and can arbitrarily change the circumstances under which one is safe from intolerance (Verkuyten et al., 2020b, 2020a). Nevertheless, the bounded freedom afforded by being tolerated is considerably more enabling than the rigid exclusion and rejection experienced in discrimination.

To summarize, tolerance can be related to lower well‐being because it threatens several social identity needs among targets, such as self‐esteem, belonging, and a sense of control. Tolerance does not involve the negative behavioural interference that characterizes discrimination, but nonetheless casts targets as deviant and subordinate to more powerful others (Verkuyten et al., 2020b, 2020a). Deleterious consequences can therefore be expected with regard to emotions, life satisfaction, self‐esteem, and a sense of control. Thus, we expect being tolerated to have negative implications for well‐being outcomes in comparison with being accepted, but less so compared to being discriminated against.

### Overview of studies

We conducted a survey and two experiments to test our predictions regarding the link between being tolerated and well‐being. Survey participants in Study 1 answered questions about the frequency of experiences with being tolerated, as well as being discriminated against or being accepted. In Study 2, participants were randomly assigned to a toleration condition (versus discrimination and acceptance conditions) to examine the causal impact of recalling experiences of being tolerated on well‐being. In Study 3, participants engaged with vignettes about being tolerated (compared to discriminated against and being accepted) to examine if this engagement triggered feelings of social identity threat that are subsequently related to lower well‐being.

Across all studies, we focused on participants’ psychological well‐being through a range of indexes, including negative but also positive emotions (which constitute separate dimensions; Diener & Emmons, 1984), life satisfaction, sense of control, and self‐esteem. First, we expected that higher perceived tolerance is independently related to lower well‐being (H1). Second, the experience of being tolerated is expected to have a more negative association with well‐being than being accepted, but a less negative impact than being discriminated against (H2). Third, we expected that the negative well‐being effects of being tolerated (compared to discrimination and acceptance) are due to threatened social identity needs (H3).

All studies were conducted in the United States among racial/ethnic minorities and took trait‐like negative emotionality into account (Tellegen & Waller, 2008; Watson & Clark, 1984), because this predisposition might inflate the link between perceived negative group treatment and well‐being (Lilienfeld, 2017). Trait‐like negative emotionality can lead to exaggerated perceptions of unfair treatment in ambiguous circumstances (such as being tolerated; Verkuyten et al., 2020b, 2020a) and can therefore be an important confound in research studying the relation between perceived negative treatment and well‐being (Ong, Burrow, Fuller‐Rowell, Ja, & Sue, 2013).

## STUDY 1

In Study 1, we examined the relations between being tolerated and minority well‐being, compared to being accepted and discriminated against. We examined five distinct components of well‐being (positive affect, negative affect, self‐esteem, life satisfaction, and sense of control in life) as two higher‐order latent factors encapsulating positive and negative well‐being, respectively.

### Method

#### Participants

A total of 330 non‐white racial/ethnic minorities in the United States were retained for analysis.^1^ Of these, 182 respondents self‐identified as African American, 44 as Hispanic or Latinx, 53 as Asian American, 16 as Native American or Alaska Natives, 17 as multiracial American, 2 as Arab or Middle Eastern American, and 16 reported other (non‐white) racial or ethnic backgrounds. Their ages ranged from 17 to 73 years old (*M* = 37.0, *SD* = 14.4), and the sample was 72.7% female. On a 9‐point scale for level of education, ranging from ‘no school’ to ‘doctorate degree’, the median education level was ‘some college’. On an 8‐point income scale, ranging from ‘under $10,000’ to ‘$200,000 or more’, the median income was between $25,000 and $49,999. On average, the sample leaned politically liberal on a 7‐point scale ranging from 1 = very liberal to 7 = very conservative (*M* = 3.41, *SD* = 1.91).

#### Procedure

Respondents were reached through Qualtrics’s online market research panels in December 2018. Respondents first reported their ethnic/racial background, and those who identified as white or European American were filtered out, with a 20–30% response rate for non‐white participants. Participants then reported their age, sex, education level, income, and political orientation. Subsequently, they answered the main survey questions described below and finally answered a suspicion probe. Unless otherwise stated, all questions and measures used 7‐point Likert‐type scales. Upon completion of the survey, the participants were fully debriefed and received a small financial reward. The research (three studies) was approved by the Faculty Ethics Review Board of the Faculty of Social and Behavioural Sciences at Utrecht University.

### Measures

#### Perceived toleration, acceptance, and discrimination

Participants answered a series of questions about the frequency of their being tolerated, discriminated against, and accepted, in counterbalanced order. Because we wanted to compare the three types of experiences, we used the same measure for all three by asking about experiences in different social contexts directly, rather than focusing on particular forms of discrimination or tolerance (e.g., Operario & Fiske, 2001; Stronge et al., 2016). Participants indicated how often they felt tolerated, discriminated against, or accepted because of their race or ethnicity across seven social contexts: at work, at school, during leisure activities, at clubs or organizations, in their neighbourhood, on social media, and overall (1 = never, 7 = always).

Each set of questions was preceded by a description of what being tolerated, discriminated against, or accepted meant. For being tolerated, the description read: ‘Situations in which people put up with your cultural beliefs or practices. For example, when you get the sense that other people have objections to the norms, practices, or way of life of your racial or ethnic group, but they nevertheless do not interfere with what you are doing. Have you ever experienced people putting up with you despite objections to the norms, practices, or way of life of your ethnic or racial group?’. For discrimination, the description read: ‘Situations in which you are treated unfairly because of the norms, practices, or way of life of your racial or ethnic group. For example, when other people exclude you or treat you unjustly based on your race or ethnicity. Have you ever experienced unfair treatment because of your race or ethnicity?’. For acceptance, the description read: ‘Situations in which you are welcomed and people are genuinely appreciative of the norms, practices, or way of life of your racial or ethnic group. For example, when people welcome the perspective of your ethnic or racial group and help you feel accepted. Have you ever experienced being welcomed because of your race or ethnicity?’.

#### Well‐being

##### Positive and negative affect

We administered a 15‐item version of the Positive and Negative Affect Schedule (Watson, Clark, & Tellegen, 1988), which asked participants to what extent they are currently experiencing seven positive emotions (e.g., happy, comfortable, and proud) and eight negative emotions (e.g., scared, irritable, and downhearted). A confirmatory factor analysis supported a two‐factor model with separate factors for positive and negative affect, χ^2^(76) = 248.806, *p* < .001; CFI = .934; RMSEA = .083, 90% CIs [0.072, 0.095]; SRMR = .056, over a one‐factor model, χ^2^(77) = 1,040.246, *p* < .001; CFI = .633; RMSEA = .195, 90% CIs [0.184, 0.205]; SRMR = 0.151. One item (‘bold’) was removed due to low loading (<0.6), and the residuals of two positive items (‘happy’ and ‘cheerful’) were covaried. Both scales had good internal consistency, α_positive_ = .89 and α_negative_ = .91.

##### Life satisfaction

We used the Satisfaction with Life Scale (Diener, Emmons, Larsen, & Griffin, 1985), which consists of five items (e.g., ‘In most ways my life is close to ideal’; α = .87).

##### Self‐esteem

We measured personal self‐esteem with a single‐item measure (‘I have high self‐esteem’; Robins, Hendin, & Trzesniewski, 2001) which we treated as latent by estimating its error variance and multiplying the variance of the single indicator with the unreliability of the Rosenberg Self‐Esteem Scale (1965).

##### Lack of control

We measured participants’ perceived lack of control using Lachman and Weaver’s (1998) scale consisting of eight items (e.g., ‘I have little control over the things that happen to me’), although the three reverse‐coded items were dropped due to factor loadings below 0.6. The remaining scale had good internal consistency (α = .82).

##### Negative emotionality

We used eight items from the Negative Emotionality subscale of Tellegen’s (1982) Multidimensional Personality Questionnaire (e.g., ‘Many people try to push me around’). One item (‘People rarely try to take advantage of me’) was dropped due to low factor loading, with the resulting scale having good internal consistency, α = .87.

##### Higher‐order well‐being factors

In the interest of parsimony, we combined the well‐being measures into two second‐order factors: one for positive well‐being, consisting of positive affect, self‐esteem, and life satisfaction, and one for negative well‐being, consisting of negative affect and lack of control. We freed the covariance of the positive and negative affect scales because they shared common method variance. The resulting model had an acceptable fit, χ^2^(268) = 564.67, *p* < .001; CFI = .932; RMSEA = .058, 90% CIs [0.051, 0.065]; SRMR = .061^2^.

## Results

### Distinguishing between being tolerated, discriminated against, and accepted

Using Mplus 8.0, we examined whether perceived discrimination, toleration, and acceptance are empirically distinguishable experiences. We used an adaptation of the multi‐trait‐multi‐method approach by taking participants’ general experiences within a particular context (e.g., at school, at work) into account. Specifically, we allowed the residuals of discrimination, toleration, and acceptance items to correlate within the same social context to account for common method variance (i.e., residuals to items pertaining to school, work, etc.). Table 1 shows the fit indices of the models we tested. The one‐factor model (Model 1) and the two‐factor combinations (Models 2‐4) did not fit the data well, while the three‐factor model (Model 5) fit the data well, indicating that the three experiences are empirically distinct.

**Table 1:** Model fit indices for perceived discrimination, toleration, and acceptance factors

Model	Chi‐square	df	CFI	RMSEA [90% CIs]	SRMR	AIC
1	3,802.89***	168	0.465	.256 [0.249, 0.263]	.219	26,162.499
2	2,380.81***	167	0.674	.200 [0.193, 0.208]	.179	24,742.412
3	2,175.23***	167	0.705	.191 [0.184, 0.198]	.219	24,536.832
4	1,770.25***	167	0.764	.171 [0.163, 0.178]	.109	24,131.858
5	398.957***	165	0.966	.066 [0.057, 0.074]	.033	22,764.565

Notes

AIC = Akaike's information criterion; CFI = comparative fit index; CIs = confidence intervals; df = degrees of freedom; RMSEA = root‐mean‐square error of approximation; SRMR = standardized root‐mean‐square residual.

Model 1 was a one‐factor model. Models 2 through 4 were two‐factor models where either discrimination, toleration, or acceptance, respectively, stood alone while the items of the other two constructs were made to load on the same factor. Model 5 was a three‐factor model.

*** *p* < .001.

The correlations between perceived toleration, discrimination, and acceptance (Table 2) also indicate that these are distinct experiences. Perceived toleration had a moderate positive correlation with perceived discrimination, and a weaker positive association with perceived acceptance, *z* = 3.81, *p* < .001^3^. Thus, participants seemed to understand being tolerated in a somewhat negative light, as more similar to being discriminated against.

**Table 2:** Descriptive statistics and zero‐order correlations between focal variables in Study 1

Variable	*M*	*SD*	1	2	3	4	5	6	7	8
1. Perceived discrimination	3.09	1.65	–							
2. Perceived tolerance	3.24	1.79	.52**	–						
3. Perceived acceptance	4.29	1.79	.12*	.29**	–					
4. Life satisfaction	4.24	1.52	‒.08	‒.34	.16**	–				
5. Self‐esteem	4.84	1.86	.02	.02	.18**	.56**	–			
6. Positive affect	4.24	1.26	‒.01	.05	.18**	.50**	.37**	–		
7. Negative affect	2.76	1.44	.30**	.27***	‒.09	‒.25**	‒.21**	‒.38**	–	
8. Lack of control	3.39	1.51	.28**	.18**	‒.01	‒.20**	‒.27**	‒.13*	.40**	–
9. Negative emotionality	3.55	1.51	.40**	.28**	‐.01	‐.12*	‐.07	‐.04	.30**	.44**

* *p* < 05

** *p* < .01

*** *p* < .001.

A one‐way repeated‐measures ANOVA found significant and substantial differences in the prevalence of participants’ perceptions of being tolerated, discriminated against, and accepted, *F*(1.80, 591.18) = 66.43, *p* < .001, η_p_^2^ = .168. Planned contrasts revealed that participants felt more accepted than tolerated and that the prevalence of perceived toleration and discrimination did not significantly differ (see Table 2).

#### Perceived group treatments and well‐being

**Figure 1:**
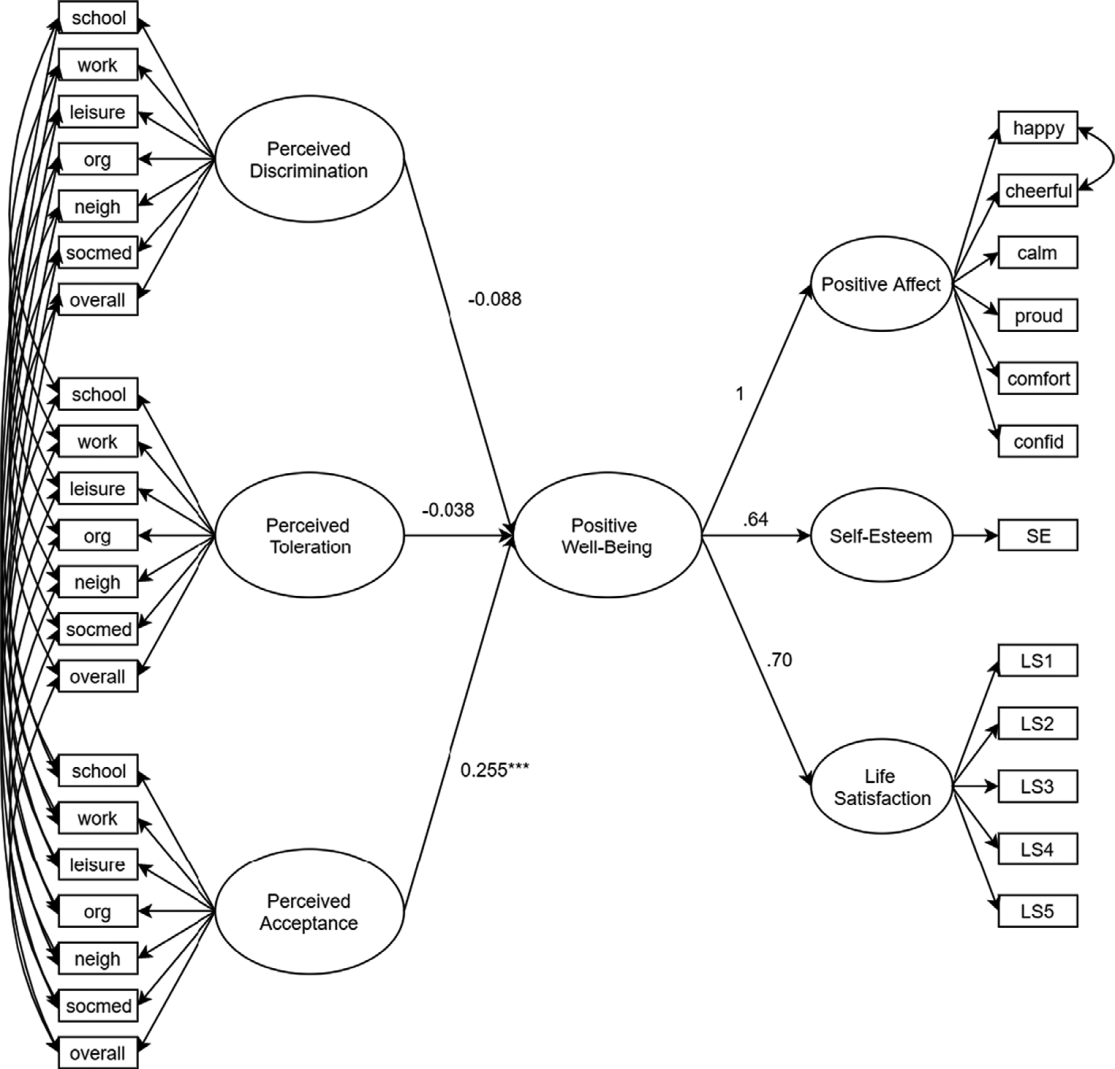
Relations of perceived group treatment to positive well‐being. *Note*: ***p < .001; comfort = comfortable; confid = confident; LS = life satisfaction; neigh = neighbourhood; org = organizations; SE = self‐esteem; socmed = social media.

**Figure 2:**
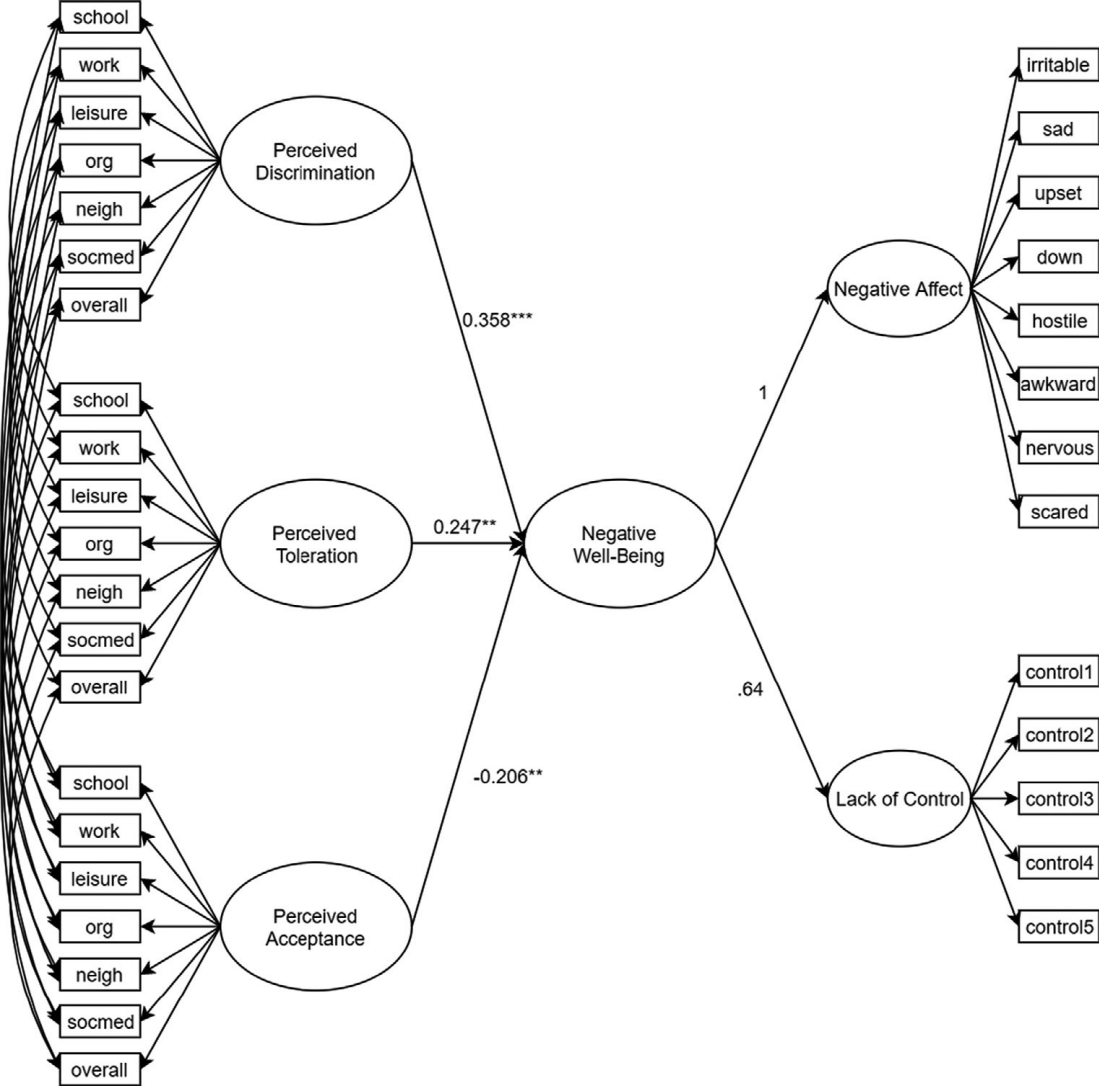
Relations of perceived group treatment to negative well‐being. *Note*: **p < .01; ***p < .001; down = downhearted; neigh = neighbourhood; org = organizations ; socmed = social media.

We created a structural model in which we specified direct paths between perceived toleration, discrimination, and acceptance (accounting for common method variance) and the second‐order factors of positive and negative well‐being (see Figures 1 and 2, respectively). The model had an acceptable fit to the data, χ^2^(952) = 1,721.915, *p* < .001; CFI = .933; RMSEA = .050, 90% CIs [0.046, 0.053]; SRMR = .051. Perceived acceptance was associated with greater positive well‐being, while the other perceived group treatments were not. In support of H1, perceived discrimination and perceived toleration were both associated with lower well‐being, while perceived acceptance was associated with higher well‐being (see Table S1 in the Appendix S1 for more information).

To test the second hypothesis, we conducted a series of Wald tests comparing the coefficients for tolerance, discrimination, and acceptance on well‐being. Concerning positive well‐being, we found that the coefficients for tolerance and discrimination did not differ, *W*(1) = 0.184, *p* = .668, whereas the coefficient for tolerance was significantly smaller than the one for acceptance, *W*(1) = 6.394, *p* = .011. The same pattern emerged for negative well‐being, such that the coefficients for tolerance and discrimination did not differ from each other, *W*(1) = 1.01, *p* = .315, but those for tolerance and acceptance differed significantly, *W*(1) = 11.497, *p* < .001. These findings partially support our second hypothesis: tolerance related differently to well‐being than acceptance, but did not differ from discrimination. We also tested whether the effects differed if we studied well‐being as five separate facets rather than as two higher‐order factors, and found that this analysis yielded the same pattern of results (see Table 3).

**Table 3:** Betas and standard errors for structural regression predicting well‐being from perceived group treatment for Study 1

Outcome	Predictor	*Β*	*SE*
Positive affect	Discrimination	‒.059	.071
	Tolerance	.011	.073
	Acceptance	.216***	.060
Negative affect	Discrimination	.222**	.065
	Tolerance	.221**	.067
	Acceptance	‒.200***	.056
Life satisfaction	Discrimination	‒.109	.070
	Tolerance	‒.044	.072
	Acceptance	.208**	.060
Self‐esteem	Discrimination	.020	.071
	Tolerance	‒.047	.073
	Acceptance	.210**	.060
Lack of control	Discrimination	.298***	.069
	Tolerance	.069	.073
	Acceptance	‒.072	.062

** *p* < .01

*** *p* < .001.

#### Controlling for negative emotionality and demographics

Next, we reran the structural regression model including trait‐like negative emotionality as a control variable. This attenuated the links between perceived discrimination, tolerance, and acceptance with negative well‐being, but did not change the association between perceived acceptance and positive well‐being (see Table S3 in the Appendix S1). Thus, the relations between group treatments and well‐being can be partially attributed to trait‐like negative emotionality, but importantly, partialling out its contribution yields a similar pattern of findings. Similarly, including demographic control variables such as sex, age, education, income, ethnicity, and political orientation reduced the strength of some coefficients, but the overall pattern of associations did not change (see Table S4 in Appendix S1).

### Discussion

Participants perceived experiences with being tolerated as distinct from both being discriminated against and being accepted (Bagci et al., in press; Cvetkovska et al., 2020). Furthermore, and in support of our first hypothesis, perceived tolerance was independently related to lower well‐being, likely due to its threat to social identity needs (Verkuyten et al., 2020b, 2020a). However, although we hypothesized that perceived tolerance would be less strongly associated with lower well‐being than perceived discrimination, we found that perceived toleration had the same relation to well‐being that perceived discrimination had. Finally, we found that accounting for individual differences in trait‐like negative emotionality did not eliminate the links between the perceived group treatments and well‐being.

## STUDY 2

The cross‐sectional findings of Study 1 suggest that experiences with being tolerated can be unsatisfactory to minority group members. In Study 2, we conducted an experiment in order to establish whether the perception of being tolerated affects (situational) well‐being. Previous research has found that being reminded of and recalling experiences of discrimination undermine well‐being (see Schmitt et al., 2014) and we expected to find this to be the case also for being tolerated. We had our participants recall either experiences of being discriminated against, being tolerated, or being accepted and measured similar well‐being outcomes as in Study 1, while also assessing the potential confounding role of trait‐like negative emotionality.

### Method

#### Participants

The participants were 315 racial/ethnic minorities in the United States. Again, the majority self‐identified as African American (185), 32 were Hispanic or Latinx (but not White/European), 39 were Asian American, 17 were Native American or Alaska Natives, 36 were multiracial American, 1 was Arab or Middle Eastern American, and 5 were from other racial/ethnic minority backgrounds. Their ages were between 18 and 83 years old (*M* = 41.2, *SD* = 16.1) and 80.3% were female. The median education level was ‘some college’ and the median income was between $25,000 and $49,999. On average, the sample leaned liberal in political views (*M* = 3.35, *SD* = 1.97).

#### Procedure and design

Similar to Study 1, data were collected through Qualtrics market research panels and yielded a similar response rate. After giving informed consent and reporting their demographic information, participants were randomly assigned to one of three experimental conditions, detailed below.

We employed a between‐subjects experimental design with three conditions using an adaptation of the questions‐as‐treatments experimental approach (e.g., Ben‐Nun Bloom, Arikan, & Courtemanche, 2015; Chong, Citrin, & Conley, 2001). In each condition and as part of the experimental manipulation, participants first answered a set of questions about how often they experienced being tolerated, accepted, or discriminated against, depending on condition (see Study 1). Subsequently, they were asked to describe a specific experience that they or someone they know^4^ has had of being either tolerated, accepted, or discriminated against. Specifically, participants were asked to remember and describe one vivid example and to take a few minutes ‘to recall and describe the situation. What happened? Who was involved? Where did it happen?’. This writing exercise (McQueen & Klein, 2006) was meant to make the experience of being tolerated, discriminated against, or accepted salient.

### Measures

Unless otherwise stated, all questions and measures used 7‐point Likert‐type scales.

#### Well‐being

##### Positive and negative affect

We administered the same 15‐item version of the Positive and Negative Affect Schedule (Watson et al., 1988) used in Study 1, with the sole difference being that we now asked participants to what extent they had felt those emotions during the experience that they had described as part of the experimental manipulation. If participants described an experience that someone else had had, they reported how they think that person would have felt at the time.

We found that positive and negative affect formed distinct scales, as a two‐factor model, χ^2^(53) = 282.30, *p* < .001, CFI = .911, RMSEA = .117, 90% CIs [0.104, 0.131], SRMR = .060, fit the data better than a one‐factor model, χ^2^(90) = 1,012.46, *p* < .001, CFI = .699, RMSEA = 0.180, 90% CIs [0.170, 0.190], SRMR = .122. Two items (‘bold’ and ‘calm’) had to be dropped from the positive affect subscale due to low loadings. Modification indices suggested covarying two other items (‘proud’ and ‘confident’). The resulting scale was highly reliable, α = .93. In the negative affect subscale, three items (‘nervous’, ‘scared’, and ‘hostile’) were dropped due to low loadings. The resulting scale had good internal consistency, α = .87. With these modifications, the model fit improved markedly, χ^2^(33) = 117.27, *p* < .001, CFI = .964, RMSEA = .090, 90% CIs [0.073, 0.108], SRMR = .040.

##### Other well‐being measures

We measured life satisfaction (α = .88), self‐esteem, and sense of lack of control (α = .83) using the same procedures we had used in Study 1. We dropped the same three items from the sense of lack of control scale as in Study 1. We also measured negative emotionality in the same way as in Study 1, this time freeing the covariances between two pairs of items to improve the model fit. The scale had good internal consistency (α = .87).

In this study, we did not group the well‐being facets into two second‐order factors, as the positive and negative affect scales pertained to the particular situation which participants reported on, whereas the other measures had a more situation‐independent scope. Because this would have resulted in too few indicators for each second‐order factor, we kept each of the five well‐being outcomes separate in the analysis.

##### Data management

We originally obtained 474 responses, but we had to exclude 159 participants from the analyses. Two were removed for being under 18. Further, given the importance to the study that participants understood the manipulation and recalled the appropriate experiences, two independent coders examined responses to the manipulation check, in which participants were asked to describe an experience of being discriminated against, being tolerated, or being accepted. Discrepancies between coders were resolved through discussion. Eighteen responses were removed from the discrimination condition: two were nonsensical, 12 preferred not to answer, one was irrelevant, and three described experiences of acceptance. Eighty responses were removed from the tolerance condition, of which three were nonsensical, 35 preferred not to answer, 26 described instances of overt discrimination, 14 were irrelevant, and two described instances of full acceptance. We removed 59 responses from the acceptance condition, of which four were nonsensical, 16 preferred not to answer, 19 described instances of overt discrimination, six were irrelevant, and 14 described instances of acceptance by one’s ingroup rather than an outgroup.

We ran a series of t‐tests and chi‐square tests to examine whether those we excluded differed from those we included in terms of their demographics. All of the t‐tests concerning age, education, income, and political orientation were non‐significant (all *t*_s_ < 1.312, all *p*_s_ > .191). There also was no association between ethnicity and gender and being included or excluded in the sample, χ^2^(6) = 2.655, *p =* .851, and χ^2^(2) = 5.904, *p =* .052, respectively. Furthermore, participants’ demographics did not differ between the three experimental conditions.

### Results

#### Relations between condition and well‐being

Using Mplus 8.0, we tested whether well‐being differed by experimental condition. We ran structural regressions with condition dummies as the independent variables (tolerance as the reference category) and the five well‐being measures (positive affect, negative affect, life satisfaction, self‐esteem, and perceived lack of control) as dependent variables. The results of these analyses can be found in Table 4, while bivariate correlations can be found in Table S5 in the Appendix S1. As expected, positive affect was lower in the tolerance condition (*M* = 3.26, *SD* = 1.90) compared to the acceptance condition (*M* = 5.84, *SD* = 1.16), but higher compared to the discrimination condition (*M* = 2.43, *SD* = 1.55), *F*(2, 312) = 146.11, *p* < .001, η_p_^2^ = .484. The pattern for negative affect was reversed, *F*(2, 312) = 124.94, *p* < .001, η_p_^2^ = .445, with the tolerance condition (*M* = 4.06, *SD* = 1.63) again being intermediate between acceptance (*M* = 2.15, *SD* = 1.34) and discrimination (*M* = 5.01, *SD* = 1.24). Life satisfaction, *F*(2, 312) = 3.30, *p* = .038, η_p_^2^ = .021, was higher in the tolerance condition (*M* = 4.53, *SD* = 1.27) than in the discrimination condition (*M* = 4.10, *SD* = 1.56). All other contrasts were non‐significant.

**Table 4:** Betas and standard errors for structural regression predicting well‐being from experimental condition

Outcome	Predictor	*Β*	*SE*
Positive affect	Discrimination	‒.200***	.048
	Acceptance	.621***	.043
Negative affect	Discrimination	.287***	.053
	Acceptance	‒.513***	.050
Life satisfaction	Discrimination	‒.132	.073
	Acceptance	.011	.073
Self‐esteem	Discrimination	‒.002	.075
	Acceptance	.045	.075
Lack of control	Discrimination	.074	.076
	Acceptance	‒.028	.076

*** *p* < .001.

#### Controlling for negative emotionality

To test the possibility that negative emotionality inflates the link between perceived group‐based treatment and well‐being detriments, we first tested whether negative emotionality differed across experimental conditions, and found that it did not, *F*(2, 312) = 0.035, *p* = .965, η_p_^2^ < .001. We then controlled for negative emotionality when predicting the five well‐being outcomes from experimental condition and found that negative emotionality was related to each of the well‐being outcomes, but its inclusion into the model did not change the original pattern of results (see Table 5). We also reran the main analysis while controlling for age, sex, ethnicity, education, income, and political orientation, and the pattern of findings was unchanged.

**Table 5:** Betas and standard errors for structural regression predicting well‐being from experimental condition while controlling for negative emotionality

Outcome	Predictor	*Β*	*SE*
Positive affect	Discrimination	‒.202***	.048
	Acceptance	.620***	.042
	Negative emotionality	.094*	.041
Negative affect	Discrimination	.286***	.053
	Acceptance	‒.514***	.050
	Negative emotionality	.116*	.046
Life satisfaction	Discrimination	‒.129	.072
	Acceptance	.012	.072
	Negative emotionality	‒.190**	.061
Self‐esteem	Discrimination	.003	.073
	Acceptance	.046	.073
	Negative emotionality	‒.264***	.060
Lack of control	Discrimination	.065	.069
	Acceptance	‒.028	.070
	Negative emotionality	.485***	.053

* *p* < 05

** *p* < .01

*** *p* < .001.

#### Additional analyses

We explored how common the perceptions of being discriminated against, tolerated, or accepted were in our sample. We ran a one‐way ANOVA and found that the means of the three perceptions were significantly different, *F*(2, 312) = 38.62, *p* < .001, η_p_^2^ = .198. Similar to Study 1, planned contrasts showed that participants in the acceptance condition reported the highest frequency of experiencing this treatment (*M* = 5.17, *SD* = 1.37, *p* < .001). The frequency of being tolerated (*M* = 3.69, *SD* = 1.68) and being discriminated against (*M* = 3.49, *SD* = 1.51) did not significantly differ (*p* = .321). With this in mind, we controlled for the frequency of experiencing discrimination, acceptance, or toleration when predicting the effects of condition on well‐being. This did not change the pattern of results presented in Table 4, and neither did controlling for the demographic variables (see Table S6 in the Appendix S1).

### Discussion

In support of H1 and H2, we found that reflecting on experiences of being tolerated leads to situationally lower well‐being than experiences involving acceptance, but higher well‐being than experiences involving discrimination. However, the experimental manipulation did not affect more stable well‐being aspects such as self‐esteem and perceived control. Similar to Study 1, we also found that trait‐like negative emotionality did not change the pattern of results.

## STUDY 3

In Studies 1 and 2, we found that being tolerated is associated with lower psychological well‐being. The theoretical literature on the consequences of being tolerated argues that the negative well‐being implications of being tolerated are due to threats to targets’ social identity needs (Verkuyten et al., 2020b, 2020a), and there is initial survey evidence in support of this proposition (Bagci et al., in press). Therefore, in our third study we aimed to experimentally test whether being tolerated (compared to being accepted and discriminated against) results in lowered well‐being because of threatened social identity needs. Additionally, in Study 3 we used a different experimental manipulation to address a limitation of Study 2. In that study, we had to exclude many responses from our analyses, which was probably due to the definition of tolerance presented to participants not being sufficiently clear. Therefore, in Study 3 we used vignettes that more clearly illustrated experiences of facing tolerance, discrimination, and acceptance, based on the theoretical literature (Verkuyten et al., 2020b, 2020a).

### Method

#### Participants

Our sample consisted of 409 participants who were members of racial/ethnic minority groups in the United States and did not complete the procedure too quickly (Leiner, 2013). We measured the same demographic variables on the same scales as in Studies 1 and 2. The racial composition was as follows: 181 self‐identified as African American, 103 as Asian American, 68 as Hispanic or Latino, 26 as mixed‐race, 10 as Native American or Alaska Natives, 5 as Middle Eastern, 3 as Pacific Islanders, and 13 from other (non‐white) ethnic or racial backgrounds. Their ages ranged from 18 to 71 years old (*N* = 407, *M* = 34.0, *SD* = 13.7). There were 185 men, 222 women, and 2 people of other genders. The median education level was ‘associate’s degree’; the median total annual household income was ‘between $25,000 and $49,999’; the median political self‐placement was ‘somewhat liberal’ (*M* = 3.31, *SD* = 1.55).

#### Procedure and design

Respondents were reached through Lucid, a professional survey panel, in August 2020. The procedure was similar to the one employed for Study 2 with participants being randomly assigned to one of three experimental conditions: acceptance, tolerance, or discrimination. Participants read a vignette and were asked to imagine themselves in the scenario. Each vignette began: ‘Imagine that you have recently started working at a new job in a company that is mostly white. The company does ‘Casual Fridays’, where employees can wear what they like to work. One Friday you come to work wearing a T‐shirt with symbols of your ethnic group. When your boss sees you, they […]’. In the acceptance condition, the ending was: ‘compliment you on your T‐shirt because they like diversity and express an interest in understanding the significance of the symbols’. In the tolerance condition, the ending was ‘show disapproval of the symbols on your T‐shirt because they see them as divisive, but nevertheless allow it to be worn at work because they believe in freedom of expression’. And in the discrimination condition, the ending was ‘tell you that they dislike your T‐shirt because they see it as too “ethnic” and tell you not to wear it to work again’.

To verify that the three experimental vignettes were equally convincing, we asked six questions at the end of the survey about how relatable, common, and realistic the scenario seemed to participants personally and to members of their ethnic/racial group. The items formed two reliable scales (α_self_ = .88; α_group_ = .90) and the vignettes were not differently convincing across conditions for both self, *F*(2, 406) = 0.864, *p* = .422, and group, *F*(2, 406) = 1.559, *p* = .212.

### Measures

Unless otherwise stated, all measures, scale reliabilities, and model fits are similar to those obtained for Studies 1 and 2.

#### Positive and negative affect

Our main dependent variable was the 15‐item PANAS which was used in Study 2. The instruction asked participants to rate how they would feel in the experimental scenario involving their boss. A two‐factor model, χ^2^(72) = 341.364, *p* < .001; CFI = .928; RMSEA = .096, 90% CIs [0.086, 0.106]; SRMR = .062, again fit better than a one‐factor model, χ^2^(77) = 1,957.002, *p* < .001; CFI = .495; RMSEA = .244, 90% CIs [0.235, 0.254]; SRMR = .222. The item ‘bold’ had to be dropped from the positive affect scale due to a low loading and three pairs of items were covaried to improve fit. The positive affect scale had good internal consistency, α = .92, as did the negative affect scale, α = .91.

#### Threatened social identity needs

We adapted the Threatened Social Identity Needs scale used by Bagci et al. (in press) to measure identity threat related to one’s ethnic or racial identity. This scale is based on Motivated Identity Construction Theory (Vignoles, 2011) and taps threats to esteem, belonging, efficacy, and certainty, each measured with two items. To this, we added two items addressing threat to distinctiveness. Each item began with the stem ‘As a member of my ethnic/racial group, the above scenario would make me feel…’ and ended with a description of one of the above‐threatened needs. For example, ‘ashamed’ and ‘negatively about myself’ tapped into self‐esteem, and ‘powerless’ and ‘unable to achieve my goals’ tapped into efficacy. An exploratory factor analysis indicated one factor explaining 69% of the variance. A follow‐up confirmatory factor analysis indicated a one‐factor model fit acceptably, χ^2^(35) = 147.007, *p* < .001; CFI = .966; RMSEA = .088, 90% CIs [0.074, 0.103]; SRMR = .026. The resulting scale had good internal consistency (α = .95). Following previous research, we focused on the role of the combined cluster of identity needs, which tend to work in concert, particularly among minority groups (e.g., Bagci et al., in press; Çelebi, Verkuyten, & Bagci, 2017; see Table S7 for correlations between subscales).

### Results

In Mplus 8.0, we ran structural regressions with condition dummies as the independent variables (with tolerance as the reference category), threatened social identity needs as the latent mediator, and positive and negative affect as the latent dependent variables. For the direct effect of experimental condition on well‐being, we found that those in the tolerance condition reported significantly lower well‐being than those in the acceptance condition, but higher well‐being than those in the discrimination condition. Specifically, compared to the tolerance condition, those in the acceptance condition reported feeling more positive affect (*β* = .322, *SE* = .053, *p* < .001) and less negative affect (*β* = −.171, *SE* = .041, *p* < .001). By contrast, those in the discrimination condition reported less positive affect (*β* = −.233, *SE* = .059, *p* < .001) and more negative affect (*β* = .115, *SE* = .045, *p* = .011) than those in the tolerance condition.

Next, we found that social identity needs were less threatened in the acceptance condition than in the tolerance condition (*β* = −.163, *SE* = .060, *p* = .006), but that there was no significant difference between the discrimination and tolerance conditions in terms of social identity threat (*β* = .006, *SE* = .060, *p* = .926). In turn, threatened social identity needs were associated with more negative affect (*β* = .734, *SE* = .035, *p* < .001), but had no association with positive affect (*β* = .010, *SE* = .059, *p* = .863). Importantly and as expected, we found that increased threat to social identity needs mediated the link between the acceptance‐tolerance contrast and increased negative affect (*β* = −.120, *SE* = .043, *p* = .006, 90% CIs [−0.239, −0.011]).

#### Negative emotionality

We also controlled for the influence of trait negative emotionality on positive and negative affect and threatened social identity needs and found a similar pattern of results, aside from a small negative association between identity threat and positive affect (see Table S8 in the Appendix S1). Similar findings were also found after controlling for demographic variables (see Table S9 in the Appendix S1).

### Discussion

In support of H2, we found that being tolerated results in more positive affect and less negative affect than being discriminated against, but less positive and more negative affect than being accepted. However, being tolerated did not differ from being discriminated against in terms of the threat it presented to social identity needs, and both situations were perceived as more threatening than being accepted. Moreover, we found that higher threat to social identity needs mediated the link between increased negative affect and being tolerated as opposed to being accepted.

### GENERAL DISCUSSION

Although tolerance is increasingly promoted as a way to negotiate intergroup differences and has been found to be a distinct response to disapproved‐of behaviours, little is known about the implications for the well‐being of tolerated minority group members. Tolerance implies forbearance with conditional permission for minority members to engage in practices of which the majority disapproves (Cohen, 2004). Being tolerated is argued to be an improvement upon being discriminated against, but is not full acceptance (Verkuyten et al., 2020b, 2020a). While it is considered desirable to be tolerant, it may not be desirable to be ‘put up with’, and describing someone as tolerable has negative connotations (Brown, 2006). Following theoretical work on the implications of being tolerated and their mechanisms (Verkuyten et al., 2020b, 2020a), we empirically examined the possible negative consequences of being tolerated for the well‐being of members of ethnic minority groups.

In Study 1, we found that being tolerated is a distinct experience from being discriminated against and being accepted (see also Bagci et al., in press; Cvetkovska et al., 2020). Furthermore, perceiving oneself to be tolerated was associated with negative well‐being, independently of perceived discrimination. Thus, being tolerated and being discriminated against were both associated with negative well‐being, whereas higher perceived acceptance was associated with lower negative well‐being. Additionally, in Studies 2 and 3 we found causal evidence that the experience of being tolerated (compared to being accepted) can situationally lower the well‐being of ethnic minorities, but not as much as being discriminated against. This pattern of results supports the theoretical proposition (Verkuyten et al., 2020b, 2020a) that being merely tolerated can be a negative experience that is intermediate between acceptance and discrimination (Scanlon, 2003).

However, tolerance is likely experienced as closer to discrimination than to full acceptance because it shares with discrimination the negative appraisal of minority practices and identity (Brown, 2006), although the two differ in that tolerance is not accompanied by negative action. In support of this, we found in all three studies that tolerance and discrimination showed similar negative associations with well‐being, which was also found among three minority groups in Turkey (Bagci et al., in press). In Study 2, the effect of thinking about an experience of being tolerated was in the opposite direction from being accepted and in the same direction as being discriminated against, and in Study 3, tolerance and discrimination both threatened social identity needs. Thus, although tolerance is distinct from both acceptance and (overt) discrimination in both its interpretation by targets and in its consequences for well‐being, it appears to have more subjective similarities with discrimination than with acceptance. This was also reflected in participants’ written responses to the tolerance prompt in Study 2, which sometimes resembled experiences of subtle discrimination (Jones, Peddie, Gilrane, King, & Gray, 2016). Future research should further explore the similarities and differences between being tolerated and facing various forms of discrimination, especially its more subtle forms, in minority targets’ experiences and interpretations. Intergroup interactions can be interpreted in quite different ways by different parties, so it is quite possible that acts of tolerance may be interpreted by targets as discriminatory (Demoulin, Leyens, & Dovidio, 2009).

To address the possibility that the link between negative group‐based treatment and well‐being might be inflated by not taking personality traits into account (Lilienfeld, 2017; Ong et al., 2013), we measured and controlled for participants’ trait‐like negative emotionality. Negative emotionality was correlated with perceived discrimination and perceived tolerance in Study 1, but including it in the statistical analyses did not eliminate the links between group treatment and well‐being in the three studies. Thus, in the present research, minority group members were negatively impacted by perceiving themselves to be treated negatively regardless of trait‐like tendencies to feel frequent negative emotions. Furthermore, the same pattern of findings was found while controlling statistically for demographic variables such as sex/gender, ethnicity, age, education, and political orientation. This indicates that negative emotionality and various demographic variables did not fully account for the finding that more frequent experiences of being tolerated and being discriminated against were associated with lower well‐being. Thus, the negative experiences of minority group members do not simply reflect an underlying predisposition to see the world in a negative way (Williams, 2020).

The current research makes a novel contribution to the psychological literature on the target’s perspective by focusing on toleration experiences and how these relate to psychological well‐being. However, a few limitations should be acknowledged. First, we have examined the well‐being implications of being tolerated among racial minorities (most of them African American) in the US context. This means that we cannot generalize the current findings to other minority groups (racial, ethnic, or otherwise) and other national contexts. Although some of the findings are similar to what was found in Turkey (Bagci et al., in press), there can be relevant group and country differences. For example, the prevalent ‘melting pot’ discourse in the United States might be less celebratory of tolerating intergroup differences compared to, say, the Netherlands, where tolerance is considered a self‐defining national virtue.

Second, we had to exclude a large number of responses in the tolerance condition of Study 2. When responding to our prompt, many participants reported experiences of discrimination, both subtle and blatant. One reason is that discrimination and tolerance share the negative evaluation of others, but differ in whether this translates in negative behaviour. While the negative behaviour makes discrimination relatively easy to detect, detection of toleration is more complex (Verkuyten et al., 2020b, 2020a). Tolerance implies holding objections to others’ practices but not negatively interfering with their behaviour, and this might be difficult to recognize and evaluate because it manifests in inaction. Qualitative research is needed to map out the various meanings that being tolerated might have, what cues help targets decide whether others are acting out of mere tolerance, and what consequences this has for targets’ well‐being and behaviour. Nonetheless, in Study 3 we were able to overcome this limitation by using vignettes and were able to conceptually replicate the relationship between tolerance and well‐being from Study 2.

Further research is needed that systematically examines or experimentally varies^5^ the meanings of tolerance and tests the relations between different conceptions of tolerance and targets’ well‐being. There are conceptualizations of tolerance where the reasons for overriding the objection to specific behaviours or values of the tolerated group are based in respect or esteem for those being tolerated (Cohen, 2004; Forst, 2012). These understandings of tolerance may be more similar to full acceptance and may therefore be more likely to be associated with positive well‐being (Cvetkovska et al., 2020).

Third, in Studies 1 and 2, the identity of the tolerator was unknown. We chose to use this method to cast a wide net of the many scenarios in which someone might experience being tolerated. However, that also means that we were unable to differentiate between tolerance experienced from specific individuals, particular groups, society as a whole, or even from fellow ingroup members. Further, we were also not able to consider the circumstances and situations in which tolerance occurred. And although we included participants from many ethnic minority backgrounds, we were unable to statistically differentiate between responses from these different backgrounds, which may affect the experiences of tolerance. Future research should examine the question of whether the importance of being tolerated for one’s well‐being depends on who the tolerator is, the circumstances under which tolerance occurs, and the specific background of the tolerated.

### Conclusion

Despite these limitations, the current research is one of the very first that investigates the possible predicament of being tolerated and its mechanisms for both positive and negative well‐being. Our results show that being tolerated can be harmful to minorities. It is somewhat better for minority well‐being than being discriminated outright, but is significantly worse than full acceptance. Tolerance is considered to be necessary for living with diversity and important for minority members’ ability to express and maintain their cultural and religious ways of life (see Verkuyten et al., 2019); however, tolerance can be experienced as condescending, conditional, and identity‐threatening and therefore should be carefully considered by policymakers in light of its potential to (unintendedly) harm the tolerated.

## Conflicts of interest

All authors declare no conflict of interest.

## Supporting information

**Appendix S1**. Additional analyses for Study 1, ethnic and national identification, and additional tables.
**Table S1**. Main analysis predicting well‐being from group treatment with higher order factors for Study 1.
**Table S2**. Main analysis of Study 1 with five well‐being outcomes controlling for demographic variables.
**Table S3**. Main analysis predicting well‐being from group treatment with higher order factors controlling for negative emotionality in Study 1.
**Table S4**. Main analysis of Study 1 with higher‐order well‐being outcomes controlling for demographic variables.
**Table S5**. Correlations between focal variables in Study 2 split by condition.
**Table S6**. Main analysis of Study 2 controlling for demographic variables.
**Table S7**. Correlations between threatened social identity needs subscales.
**Table S8**. Structural regressions on dependent variables and mediator controlling for negative emotionality in Study 3.
**Table S9**. Structural regressions on dependent variables and mediator controlling for demographics in Study 3.

## Data Availability

The data that support the findings of this study are openly available on the Open Science Framework at the following link [https://osf.io/vp5cj/?view_only=222fcf6bff54422b90698d821384ce12].

## Footnotes

^1^
Originally, we had 420 responses. However, following the recommendations of Leiner (2013) to remove participants who completed the study unreasonably quickly, we computed a relative speed index for each participant (median completion time divided by participant’s completion time) and removed responses with an index above 1.75, leaving 330 participants for analyses.

^2^
In all three studies, we measured ethnic and national identification as a possible moderator (e.g., Cvetkovska et al., 2020). Across the three studies, there was no consistent moderation by either national or ethnic identification, see Appendix for details.

^3^
Unexpectedly, perceived discrimination and acceptance were positively correlated, even after accounting for shared variance of some items. A possible reason is that the minority participants were suspicious about the motives of those who were accepting (Major et al., 2016).

^4^
Of the experiences described, 72.7% (acceptance) to 82.6% (discrimination; 74.4% for tolerance) were personal, and analysis conducted on only these responses yielded an identical pattern of findings as for the total sample.

^5^
We are grateful to an anonymous reviewer for this suggestion.
