# Supplementary material for: Being tolerated: Implications for well‐being among ethnic minorities
Source: Br J Psychol. 2021 Feb 11;112(3):781–803. doi: 10.1111/bjop.12492 (PMC8359250; doi:10.1111/bjop.12492)
Supplement: Supplementary file 1 — Appendix S1. Additional analyses for Study 1, ethnic and national identification, and additional tables. Table S1. Main analysis predicting well‐being from group treatment with higher order factors for Study 1. Table S2. Main analysis of Study 1 with five well‐being outcomes controlling for demographic variables. Table S3. Main analysis predicting well‐being from group treatment with higher order factors controlling for negative emotionality in Study 1. Table S4. Main analysis of Study 1 with higher‐order well‐being outcomes controlling for demographic variables. Table S5. Correlations between focal variables in Study 2 split by condition. Table S6. Main analysis of Study 2 controlling for demographic variables. Table S7. Correlations between threatened social identity needs subscales. Table S8. Structural regressions on dependent variables and mediator controlling for negative emotionality in Study 3. Table S9. Structural regressions on dependent variables and mediator controlling for demographics in Study 3. [file BJOP-112-781-s001.docx]

**Appendix**

Being Tolerated: Implications for Well-Being among Ethnic Minorities

Table of contents:

Additional Study 1 Analyses………………………………………………………………………2

Ethnic and National Identification…………………………………………………………………4

Additional Tables……………………………………………………………………….………….8

References…………………………………………………………………………………………17

**Additional Study 1 Analyses**

We re-ran our main structural regression model with five separate well-being facets (positive affect, negative affect, life satisfaction, self-esteem, and lack of control) to more closely examine which facets relate the most strongly to the perceived group treatments (Table 3). All variables in the analyses were latent and were entered simultaneously in the model, χ^2^(939) = 1651.622, *p* < .001; CFI = 0.938; RMSEA = 0.048, 90% CIs [0.044, 0.052]; SRMR = 0.048. Higher perceived discrimination and perceived tolerance were both associated with stronger negative affect, in support of the first hypothesis. Acceptance was associated with more positive affect and less negative affect, more life satisfaction, and higher self-esteem. Perceived discrimination was associated with a lesser sense of control. The sizes of the correlation coefficients for perceived discrimination and negative affect and perceived tolerance and negative affect were virtually identical (*z* = 0.01, *p* = .992), in partial support of our second hypothesis.

To test H2, we again ran a series of Wald tests for each of the five well-being outcomes comparing each coefficient for perceived tolerance to the coefficients for discrimination and acceptance. For positive affect, there was a close to significant difference between the coefficients for tolerance and acceptance, *W*(1) = -0.178, *p* = .059. For negative affect, life satisfaction, and self-esteem, there were significant differences between the coefficients for tolerance and acceptance, *W*(1) = 0.350, *p* < .001; *W*(1) = -0.193, *p* = .022; and *W*(1) = -0.254, *p* = .020, respectively. For lack of control, the coefficients for discrimination and tolerance were significantly different, *W*(1) = 0.197, *p* = .045. In general, perceived tolerance was more similar to discrimination in terms of its associations with some facets of well-being except in the case of lack of control, in partial support of H2.

We repeated the main analyses controlling for a range of demographic variables: sex (dummy coded), age, education, income, and ethnicity (dummy coded), as well as political orientation. We did this both for the higher-order well-being outcomes (see Table A1 in Appendix A) and for the five separate facets (see Table 2 in Appendix A). Some coefficients for the focal variables were reduced in strength, but the overall pattern of results remained the same.

**Ethnic and National Identification**

In addition to the other reported measures, we also examined the moderating role of ethnic and national identification on the relation between being discriminated, tolerated, and accepted on positive and negative well-being. Below is the theoretical reason for including this along with findings relating to these that are an extension on the main manuscript.

**Group Identification**

According to Social Identity Theory (Tajfel & Turner, 1979) the extent to which a social identity is central to a person’s sense of self can modulate one’s reactions to in-group relevant phenomena such as perceiving one’s social group to be devalued and dependent (Ellemers, Spears, & Doosje, 2002). For high identifiers, a threat to the in-group is a threat to the self, which can manifest as lowered self-esteem, anxiety and depression. Low identifiers, on the other hand, can more easily brush off negative treatment of their in-group and thus shield their personal self-esteem (McCoy & Major, 2003). We propose to extend this reasoning about the sensitizing role of group identification to the perception of being tolerated (e.g., McCoy & Major, 2003; Operario & Fiske, 2001). Those who identify strongly with the tolerated in-group might feel the negative effects of being tolerated more strongly.

Additionally, we propose that identification with the national group which is shared with the tolerators may have a similar sensitizing role. Group-based treatment such as discrimination or tolerance conveys not only a judgment about the target’s minority in-group, but also a judgment about whether that group should be included in the superordinate national category. For those who consider the national category to be an important aspect of the self, cues that convey inclusion or exclusion into this category should have a stronger negative impact on well-being than those who consider the national category less important to their sense of self (Huynh, Devos, & Goldberg, 2014).

Hypothesis: We expected that the relations between the perceived group treatments and well-being will be stronger for higher, compared to lower, ethnic identifiers and national identifiers.

**Study 1**

**Method**

**Ethnic and national identification.** We measured participants’ identification with their ethnic or racial group and with America by using two sets of similar items drawn from previous work (Postmes, Haslam & Jans, 2013; Phinney & Ong, 2007), such as “I identify with [my ethnic or racial group/America]” and “I have a strong sense of belonging to [my ethnic or racial group/America]”. Each scale consisted of 4 items. A one-factor model had a poor fit to the data, χ^2^(20) = 1037.989, *p* < .001; CFI = 0.548; RMSEA = 0.393, 90% CIs [0.373, 0.413]; SRMR = 0.237, but a two-factor model showed an acceptable fit, χ^2^(19) = 91.104, *p* < .001; CFI = 0.968; RMSEA = 0.107, 90% CIs [0.086, 0.130]; SRMR = 0.025. Therefore, we created separate latent scales. Both had high internal consistency, α = .92 and .93 for ethnic and national identification respectively.

**Results**

**Moderation by ethnic and national identification.** We tested the possibility that higher ethnic and national identifiers are more sensitive to how they perceive their group to be treated, amplifying any relations of group treatment to well-being. We ran a series of moderation analyses in which the latent factors of ethnic or national identification moderated the links between group treatment and the two higher-order well-being factors. There were no significant interactions between perceived group treatment and ethnic identification (all B_s_ < 0.035, all *p*_s_ > .302) or national identification (all B_s_ < 0.042, all *p*_s_ > .148).

Because ethnic and national identification were both directly related to higher positive affect, life satisfaction, and self-esteem, we also used them as controls in the main analysis. When we did this, only the associations between acceptance and life satisfaction and self-esteem decreased in size but the overall pattern of results shown in Table 3 remained the same.

We again examined whether high ethnic and national identifiers are more sensitive to group treatment with regard to each of the five well-being facets. Because this meant running a large number of analyses, we applied a Bonferroni correction which lowered the alpha criterion to just under 0.002. None of the interaction terms were significant at this level for ethnic identification (all B_s_ < 0.081, all *p*_s_ > .106) or national identification (all B_s_ < 0.047 or 0.042, all *p*_s_ > .095).

**Study 2**

Once again, we tested whether the links between perceived group treatment and well-being are stronger for higher ethnic identifiers and higher national identifiers.

**Method**

**Ethnic and national identification.** With the same measures as in Study 1 and before the experimental manipulation, we assessed participants’ ethnic and national identification. A one-factor model had a poor fit to the data, χ^2^(20) = 1181.46, *p* < .001; CFI = 0.542; RMSEA = 0.429, 90% CIs [0.409, 0.450]; SRMR = 0.252, but a two-factor model showed an acceptable fit, χ^2^(19) = 58.840, *p* < .001; CFI = 0.984; RMSEA = 0.082, 90% CIs [0.058, 0.106]; SRMR = 0.021. Therefore we created separate latent scales. The measure of ethnic identification was highly reliable (α = .95), as was the measure of national identification (α = .94).

**Results**

**The Role of Group Identification.** We tested the possibility that higher ethnic or national identifiers would be more sensitive to any effects of group treatment on well-being. We therefore regressed each well-being outcome onto group treatment, the latent factors for ethnic and national identification, and their (latent) interactions. Both ethnic, β = 0.202, *p* < .001, and national identification, β = 0.291, *p* < .001, were related to higher self-esteem. Further, national identification was related to higher positive affect, β = 0.176, *p* = .004, and to higher life satisfaction, β = 0.359, *p* < .001. Furthermore, the pattern of findings shown in Table 4 did not change when taking both ethnic and national identifications into account. Only one interaction term reached statistical significance (*B* = -0.291, *SE* = 0.147, *p* = .048). In the discrimination condition relative to the tolerance condition, the positive main effect of high ethnic identification on self-esteem is attenuated. All other interaction terms for ethnic and national identification were non-significant.

**Study 3**

In the third study, we examined the roles of ethnic and national identification as predictors of positive and negative affect and threatened social identity needs, and as moderators of the effects of condition on the dependent variables and the mediator.

We found that national identification had a main effect on positive affect, *B* = 0.327, *SE* = 0.086, *p* < .001, but no other main effects or interactions involving national identification reached statistical significance. Ethnic identification also had a main effect on positive affect, *B* = 0.167, *SE* = 0.079, *p* = .035, as well as an interaction with each condition: in the acceptance condition, compared to the tolerance condition, high ethnic identifiers experienced an even greater boost to positive affect compared to low ethnic identifiers, *B* = 0.233, *SE* = 0.118, *p* = .048; in the discrimination condition, relative to the tolerance condition, high ethnic identifiers experienced a greater reduction in positive affect than low ethnic identifiers, *B* = -0.355, *SE* = 0.130, *p* = .007. All other main effects and interactions were non-significant.

**Additional Tables**

| Table A1 | | | |
| --- | --- | --- | --- |
| *Main analysis predicting well-being from group treatment with higher order factors for Study 1.* | | | |
| Outcome | Predictor | Β | *SE* |
| Positive Well-Being | Discrimination | -0.088 | 0.075 |
|  | Tolerance | -0.038 | 0.077 |
|  | Acceptance | 0.255*** | 0.064 |
| Negative Well-Being | Discrimination | 0.358*** | 0.080 |
|  | Tolerance | 0.247** | 0.082 |
|  | Acceptance | -0.206** | 0.069 |

*Note.* ** *p* < .01; **** p* < .001.

| Table A2 |  | | |  | | |  | | |  | | |  | |
| --- | --- | --- | --- | --- | --- | --- | --- | --- | --- | --- | --- | --- | --- | --- |
| *Main analysis of Study 1 with five well-being outcomes controlling for demographic variables* | | | | | | | | | | | | | | |
|  | Outcome variables | | | | | | | | | | | | | |
|  | Positive Affect | | Negative Affect | | | Life Satisfaction | | | Self-Esteem | | | Lack of Control | | |
| Predictors | β | SE | β | | SE | β | | SE | β | | SE | β | | SE |
| Perceived Discrimination | -0.051 | 0.069 | 0.230*** | | 0.064 | -0.114^†^ | | 0.068 | 0.042 | | 0.069 | 0.324*** | | 0.067 |
| Perceived Tolerance | 0.003 | 0.071 | 0.211** | | 0.067 | -0.041 | | 0.071 | -0.034 | | 0.072 | 0.036 | | 0.073 |
| Perceived Acceptance | 0.221*** | 0.059 | -0.201*** | | 0.056 | 0.188** | | 0.059 | 0.182** | | 0.060 | -0.062 | | 0.061 |
| Age | 0.105^†^ | 0.058 | -0.083 | | 0.055 | 0.006 | | 0.058 | 0.132* | | 0.058 | -0.056 | | 0.059 |
| Sex | -0.015 | 0.056 | 0.032 | | 0.053 | -0.018 | | 0.056 | -0.069 | | 0.056 | -0.065 | | 0.057 |
| Education Level | 0.046 | 0.066 | -0.110^†^ | | 0.063 | 0.042 | | 0.066 | 0.095 | | 0.067 | -0.093 | | 0.067 |
| Income | 0.034 | 0.066 | -0.065 | | 0.062 | 0.161* | | 0.064 | 0.007 | | 0.066 | -0.149* | | 0.066 |
| Political Orientation | 0.248*** | 0.060 | -0.111^†^ | | 0.060 | 0.236*** | | 0.060 | 0.139* | | 0.062 | 0.045 | | 0.064 |
| Ethnicity |  |  |  | |  |  | |  |  | |  |  | |  |
| Asian American | -0.066 | 0.060 | -0.058 | | 0.057 | -0.037 | | 0.060 | -0.136* | | 0.060 | -0.043 | | 0.061 |
| Latinx | 0.051 | 0.062 | 0.050 | | 0.059 | -0.062 | | 0.062 | -0.093 | | 0.062 | 0.013 | | 0.063 |
| Other Ethnicity | -0.061 | 0.058 | 0.033 | | 0.055 | -0.116* | | 0.058 | -0.076 | | 0.058 | 0.050 | | 0.059 |

*Note.* * *p* < .05; ** *p* < .01; **** p* < .001; † *p* < .1. The sample size was 330 for all analyses except those involving political orientation, where the sample was 258 due to missing values. In analyses involving ethnicity, the reference group is African Americans. In analyses involving sex, the reference group is males.

| Table A3 |  |  |  |
| --- | --- | --- | --- |
| *Main analysis predicting well-being from group treatment with higher order factors controlling for negative emotionality in Study 1.* | | | |
| Outcome | Predictor | Β | SE |
| Positive Well-Being | Discrimination | -0.036 | 0.081 |
|  | Tolerance | -0.018 | 0.078 |
|  | Acceptance | 0.242*** | 0.064 |
|  | Negative Emotionality | -0.132 | 0.073 |
| Negative Well-Being | Discrimination | 0.191* | 0.081 |
|  | Tolerance | 0.145 | 0.082 |
|  | Acceptance | -0.132 | 0.068 |
|  | Negative Emotionality | 0.511*** | 0.072 |

*Note.* * *p* < .05; **** p* < .001.

| Table A4 | | | | |
| --- | --- | --- | --- | --- |
| *Main analysis of Study 1 with higher-order well-being outcomes controlling for demographic variables.* | | | | |
| Outcome | Positive Well-Being | | Negative Well-Being | |
| Predictor | β | SE | β | SE |
| Perceived Discrimination | -0.070 | 0.074 | 0.388*** | 0.080 |
| Perceived Tolerance | -0.036 | 0.076 | 0.210* | 0.084 |
| Perceived Acceptance | 0.259*** | 0.064 | -0.217** | 0.070 |
| Age | 0.086 | 0.063 | -0.106 | 0.068 |
| Sex | -0.041 | 0.060 | -0.007 | 0.066 |
| Education Level | 0.075 | 0.071 | -0.151* | 0.077 |
| Income | 0.113 | 0.070 | -0.143 | 0.077 |
| Political Orientation | 0.275*** | 0.064 | -0.079 | 0.077 |
| Ethnicity |  |  |  |  |
| Asian American | -0.097 | 0.064 | -0.075 | 0.069 |
| Latinx | -0.054 | 0.066 | 0.053 | 0.072 |
| Other Ethnicity | -0.120 | 0.062 | 0.057 | 0.068 |

*Note.* * *p* < .05; ** *p* < .01; **** p* < .001. The sample size was 330 for all analyses except those involving political orientation, where the sample was 258 due to missing values. In analyses involving ethnicity, the reference group is African Americans. In analyses involving sex, the reference group is males.

| \| Table A5 \| \| \| \| \| \| \| \| \| \| \| --- \| --- \| --- \| --- \| --- \| --- \| --- \| --- \| --- \| --- \| \| *Correlations between focal variables in Study 2 split by condition.* \| \| \| \| \| \| \| \| \| \| \|  \| M \| SD \| 1 \| 2 \| 3 \| 4 \| 5 \| 6 \| 7 \| \| Discrimination (*N* = 138) \|  \|  \|  \|  \|  \|  \|  \|  \|  \| \| 1. Ethnic identification \| 5.68 \| 1.58 \| - \|  \|  \|  \|  \|  \|  \| \| 1. National identification \| 5.45 \| 1.72 \| .295*** \| - \|  \|  \|  \|  \|  \| \| 1. Positive affect \| 2.43 \| 1.55 \| .065 \| .168* \| - \|  \|  \|  \|  \| \| 1. Negative affect \| 5.01 \| 1.24 \| .019 \| .040 \| -.285** \| - \|  \|  \|  \| \| 1. Life satisfaction \| 4.10 \| 1.56 \| .133 \| .287** \| .359*** \| -.203* \| - \|  \|  \| \| 1. Self-esteem \| 5.29 \| 1.77 \| .125 \| .337*** \| .199* \| -.006 \| .493*** \| - \|  \| \| 1. Lack of control \| 3.47 \| 1.53 \| -.030 \| -.074 \| .164 \| .134 \| -.057 \| -.214* \| - \| \| 1. Negative emotionality \| 3.53 \| 1.49 \| -.080 \| -.123 \| .049 \| .093 \| -.168* \| -.257** \| .422*** \| \| Tolerance (*N* = 78) \|  \|  \|  \|  \|  \|  \|  \|  \|  \| \| 1. Ethnic identification \| 5.53 \| 1.71 \| - \|  \|  \|  \|  \|  \|  \| \| 1. National identification \| 5.30 \| 1.69 \| .354** \| - \|  \|  \|  \|  \|  \| \| 1. Positive affect \| 3.26 \| 1.90 \| .075 \| .114 \| - \|  \|  \|  \|  \| \| 1. Negative affect \| 4.06 \| 1.63 \| .176 \| .044 \| -.269* \| - \|  \|  \|  \| \| 1. Life satisfaction \| 4.53 \| 1.27 \| .270* \| .369** \| .342** \| .029 \| - \|  \|  \| \| 1. Self-esteem \| 5.29 \| 1.51 \| .398** \| .363** \| .172 \| .049 \| .528** \| - \|  \| \| 1. Lack of control \| 3.24 \| 1.46 \| -.267* \| -.168 \| .123 \| .148 \| -.109 \| -.266* \| - \| \| 1. Negative emotionality \| 3.47 \| 1.41 \| -.060 \| -.145 \| .331** \| .110 \| -.165 \| -.070 \| .263* \| \| Acceptance (*N* = 99) \|  \|  \|  \|  \|  \|  \|  \|  \|  \| \| 1. Ethnic identification \| 6.09 \| 1.40 \| - \|  \|  \|  \|  \|  \|  \| \| 1. National identification \| 5.84 \| 1.45 \| .407** \| - \|  \|  \|  \|  \|  \| \| 1. Positive affect \| 5.84 \| 1.16 \| .137 \| .317** \| - \|  \|  \|  \|  \| \| 1. Negative affect \| 2.15 \| 1.34 \| -.078 \| -.079 \| -.319** \| - \|  \|  \|  \| \| 1. Life satisfaction \| 4.56 \| 1.68 \| .180 \| .382** \| .342** \| -.016 \| - \|  \|  \| \| 1. Self-esteem \| 5.44 \| 1.53 \| .429** \| .344** \| .458** \| -.105 \| .516** \| - \|  \| \| 1. Lack of control \| 3.17 \| 1.62 \| -.110 \| -.178 \| -.167 \| .409** \| -.284** \| -.397** \| - \| \| 1. Negative emotionality \| 3.49 \| 1.61 \| -.136 \| .089 \| -.031 \| .253* \| -.235* \| -.208* \| .483** \| \| *Note.* * *p* < .05; ** *p* < .01; **** p* < .001. \| \| \| \| \| \| \| \| \| \| | | | | | | | | | | | | | | | |
| --- | --- | --- | --- | --- | --- | --- | --- | --- | --- | --- | --- | --- | --- | --- | --- | --- | --- | --- | --- | --- | --- | --- | --- | --- | --- | --- | --- | --- | --- | --- | --- | --- | --- | --- | --- | --- | --- | --- | --- | --- | --- | --- | --- | --- | --- | --- | --- | --- | --- | --- | --- | --- | --- | --- | --- | --- | --- | --- | --- | --- | --- | --- | --- | --- | --- | --- | --- | --- | --- | --- | --- | --- | --- | --- | --- | --- | --- | --- | --- | --- | --- | --- | --- | --- | --- | --- | --- | --- | --- | --- | --- | --- | --- | --- | --- | --- | --- | --- | --- | --- | --- | --- | --- | --- | --- | --- | --- | --- | --- | --- | --- | --- | --- | --- | --- | --- | --- | --- | --- | --- | --- | --- | --- | --- | --- | --- | --- | --- | --- | --- | --- | --- | --- | --- | --- | --- | --- | --- | --- | --- | --- | --- | --- | --- | --- | --- | --- | --- | --- | --- | --- | --- | --- | --- | --- | --- | --- | --- | --- | --- | --- | --- | --- | --- | --- | --- | --- | --- | --- | --- | --- | --- | --- | --- | --- | --- | --- | --- | --- | --- | --- | --- | --- | --- | --- | --- | --- | --- | --- | --- | --- | --- | --- | --- | --- | --- | --- | --- | --- | --- | --- | --- | --- | --- | --- | --- | --- | --- | --- | --- | --- | --- | --- | --- | --- | --- | --- | --- | --- | --- | --- | --- | --- | --- | --- | --- | --- | --- | --- | --- | --- | --- | --- | --- | --- | --- | --- | --- | --- | --- | --- | --- | --- | --- | --- | --- | --- | --- | --- | --- | --- | --- | --- | --- | --- | --- | --- | --- | --- | --- | --- | --- | --- | --- | --- | --- | --- | --- | --- | --- | --- | --- | --- | --- | --- | --- | --- | --- | --- | --- | --- | --- | --- | --- | --- | --- | --- | --- | --- | --- | --- | --- | --- | --- | --- | --- | --- | --- | --- | --- | --- | --- | --- | --- | --- | --- | --- | --- | --- | --- | --- | --- | --- | --- | --- | --- | --- | --- | --- | --- | --- | --- | --- | --- | --- |
| Table A6 |  | | |  | | |  | | |  | | |  | |  |
| *Main analysis of Study 2 controlling for demographic variables* | | | | | | | | | | | | | | |  |
|  | Outcome variables | | | | | | | | | | | | | |  |
|  | Positive Affect | | Negative Affect | | | Life Satisfaction | | | Self-Esteem | | | Lack of Control | | |  |
| Predictors | β | SE | β | | SE | β | | SE | β | | SE | β | | SE |  |
| Discrimination vs Tolerance | -0.180*** | 0.050 | 0.332*** | | 0.055 | -0.155 | | 0.073 | -0.001 | | 0.075 | 0.130^†^ | | 0.077 |  |
| Acceptance vs Tolerance | 0.638*** | 0.044 | -0.483*** | | 0.053 | 0.018 | | 0.073 | 0.061 | | 0.075 | 0.027 | | 0.077 |  |
| Age | 0.039 | 0.043 | -0.032 | | 0.048 | 0.126* | | 0.061 | 0.201** | | 0.062 | -0.188** | | 0.064 |  |
| Sex | -0.069^†^ | 0.041 | -0.025 | | 0.046 | -0.120* | | 0.060 | -0.036 | | 0.061 | -0.157* | | 0.062 |  |
| Education Level | 0.038 | 0.045 | 0.024 | | 0.050 | 0.124^†^ | | 0.065 | 0.066 | | 0.066 | -0.012 | | 0.068 |  |
| Income | -0.084^†^ | 0.045 | -0.025 | | 0.050 | 0.101 | | 0.065 | 0.027 | | 0.066 | -0.015 | | 0.068 |  |
| Political Orientation | 0.016 | 0.041 | 0.013 | | 0.047 | 0.137* | | 0.060 | 0.084 | | 0.061 | -0.041 | | 0.063 |  |
| Ethnicity |  |  |  | |  |  | |  |  | |  |  | |  |  |
| African American | -0.010 | 0.042 | 0.105* | | 0.047 | 0.057 | | 0.061 | 0.157* | | 0.062 | -0.060 | | 0.064 |  |

*Note.* * *p* < .05; ** *p* < .01; **** p* < .001; † *p* < .1. In analyses involving ethnicity, the reference group is non-African Americans. In analyses involving sex, the reference group is males.

| Table A7 | | | | | | | |
| --- | --- | --- | --- | --- | --- | --- | --- |
| *Correlations between threatened social identity needs subscales.* | | | | | | | |
|  | M | SD | 1 | 2 | 3 | 4 |  |
| 1. Esteem | 2.78 | 1.81 | - |  |  |  |  |
| 2. Belonging | 3.42 | 1.88 | .749*** | - |  |  |  |
| 3. Efficacy | 3.09 | 1.82 | .804*** | .819*** | - |  |  |
| 4. Certainty | 2.84 | 1.83 | .815*** | .735*** | .781*** | - |  |
| 5. Distinctiveness | 3.26 | 1.75 | .740*** | .713*** | .751*** | .702*** |  |

*Note.* **** p* < .001; The scales were created by averaging the items within each and running bivariate correlations.

| Table A8 | | | | | | | | | |  |
| --- | --- | --- | --- | --- | --- | --- | --- | --- | --- | --- |
| *Structural regressions on dependent variables and mediator controlling for negative emotionality in Study 3* | | | | | | | | | |  |
| Outcome variables | | | | | | | | | |  |
|  | Positive Affect | | | Negative Affect | | | Threatened Social Identity Needs | | | |
| Predictors | | β | SE | | β | SE | | β | SE |  |
| Threatened social identity needs | | -0.123* | 0.060 | | 0.674*** | 0.044 | |  |  |  |
| Acceptance vs. Tolerance | | 0.319*** | 0.050 | | -0.172*** | 0.041 | | -0.125* | 0.053 |  |
| Discrimination vs. Tolerance | | -0.218*** | 0.056 | | 0.123** | 0.044 | | 0.032 | 0.052 |  |
| Negative Emotionality | | 0.260*** | 0.058 | | 0.121** | 0.046 | | 0.500*** | 0.052 |  |

*Note.* * *p* < .05; ** *p* < .01; **** p* < .001

| Table A9 | | | | | | |
| --- | --- | --- | --- | --- | --- | --- |
| *Structural regressions on dependent variables and mediator controlling for demographics in Study 3* | | | | | | |
| Outcome Variables | | | | | | |
|  | Positive Affect | | Negative Affect | | Threatened Social Identity Needs | |
| Predictors | β | SE | β | SE | β | SE |
| Threatened Social Identity Needs | -0.033 | 0.054 | 0.727*** | 0.036 |  |  |
| Acceptance vs. Tolerance | 0.295*** | 0.054 | -0.170*** | 0.043 | -0.165** | 0.059 |
| Discrimination vs. Tolerance | -0.219*** | 0.055 | 0.116* | 0.045 | 0.011 | 0.058 |
| Age | -0.041 | 0.046 | -0.079* | 0.036 | -0.072 | 0.047 |
| Gender | 0.111* | 0.045 | -0.007 | 0.035 | 0.004 | 0.051 |
| Education Level | 0.050 | 0.058 | 0.090* | 0.042 | 0.079 | 0.060 |
| Income | -0.147** | 0.053 | -0.002 | 0.042 | -0.134* | 0.059 |
| Political Orientation | 0.181*** | 0.048 | -0.031 | 0.041 | 0.185** | 0.059 |
| Ethnicity |  |  |  |  |  |  |
| Asian American | -0.108* | 0.050 | 0.030 | 0.037 | 0.097^†^ | 0.055 |
| Latinx | -0.060 | 0.047 | -0.031 | 0.039 | -0.017 | 0.054 |
| Mixed Race | -0.068 | 0.041 | -0.007 | 0.033 | -0.007 | 0.054 |
| Other Ethnicity | -0.076 | 0.051 | 0.015 | 0.046 | 0.057 | 0.041 |

*Note.* * *p* < .05; ** *p* < .01; **** p* < .001; In analyses involving ethnicity, the reference group is African Americans; In analyses involving ethnicity, the reference group is men.

**References**

Ellemers, N., Spears, R., & Doosje, B. (2002). Self and social identity. *Annual Review of Psychology, 53*, 161-186. doi: 10.1146/annurev.psych.53.100901.135228

Huynh, Q. L., Devos, T., & Goldberg, R. (2014). The role of ethnic and national identifications in perceived discrimination for Asian Americans: Toward a better understanding of the buffering effect of group identifications on psychological distress. *Asian American Journal of Psychology, 5*, 161-171. doi: 10.1037/a0031601

McCoy, S. K., & Major, B. (2003). Group identification moderates emotional responses to perceived prejudice. *Personality and Social Psychology Bulletin, 29*, 1005-1017. doi: 10.1177/0146167203253466

Operario, D., & Fiske, S. T. (2001). Ethnic identity moderates perceptions of prejudice: Judgments of personal versus group discrimination and subtle versus blatant bias. *Personality and Social Psychology Bulletin, 27*, 550-561. doi: 10.1177/0146167201275004

Phinney, J. S., & Ong, A. D. (2007). Conceptualization and measurement of ethnic identity: Current status and future directions. *Journal of Counseling Psychology, 54*, 271-281. doi: 10.1037/0022-0167.54.3.271

Postmes, T., Haslam, S. A., & Jans, L. (2013). A single-item measure of social identification: Reliability, validity, and utility. *British Journal of Social Psychology, 52*, 597-617. doi:10.1111/bjso.12006

Tajfel, H., & Turner, J. C. (1979). An integrative theory of intergroup conflict. In W.G. Austin & S.Worchel (Eds.), *The social psychology of intergroup relations* (pp. 33–47). Monterey, CA: Brooks-Cole.
